# MiRNA-Seq reveals key MicroRNAs involved in fat metabolism of sheep liver

**DOI:** 10.3389/fgene.2023.985764

**Published:** 2023-03-09

**Authors:** Xiaojuan Fei, Meilin Jin, Zehu Yuan, Taotao Li, Zengkui Lu, Huihua Wang, Jian Lu, Kai Quan, Junxiang Yang, Maochang He, Tingpu Wang, Yuqin Wang, Caihong Wei

**Affiliations:** ^1^ Institute of Animal Science, Chinese Academy of Agricultural Sciences, Beijing, China; ^2^ Joint International Research Laboratory of Agriculture and Agri-Product Safety of Ministry of Education, Yangzhou University, Yangzhou, China; ^3^ Lanzhou Institute of Husbandry and Pharmaceutical Sciences, Chinese Academy of Agricultural Sciences, Lanzhou, China; ^4^ National Animal Husbandry Service, Beijing, China; ^5^ College of Animals Science and Technology, Henan University of Animal Husbandry and Economy, Zhengzhou, China; ^6^ Gansu Institute of Animal Husbandry and Veterinary Medicine, Pingliang, China; ^7^ College of Bioengineering and Biotechnology, TianShui Normal University, Tianshui, China; ^8^ College of Animals Science and Technology, Henan University of Science and Technology, Luoyang, China

**Keywords:** miRNA, liver, fat metabolism, Hu sheep, Tibetan sheep

## Abstract

There is a genetic difference between Hu sheep (short/fat-tailed sheep) and Tibetan sheep (short/thin-tailed sheep) in tail type, because of fat metabolism. Previous studies have mainly focused directly on sheep tail fat, which is not the main organ of fat metabolism. The function of miRNAs in sheep liver fat metabolism has not been thoroughly elucidated. In this study, miRNA-Seq was used to identify miRNAs in the liver tissue of three Hu sheep (short/fat-tailed sheep) and three Tibetan sheep (short/thin-tailed sheep) to characterize the differences in fat metabolism of sheep. In our study, Hu sheep was in a control group, we identified 11 differentially expressed miRNAs (DE miRNAs), including six up-regulated miRNAs and five down-regulated miRNAs. Miranda and RNAhybrid were used to predict the target genes of DE miRNAs, obtaining 3,404 target genes. A total of 115 and 67 GO terms as well as 54 and 5 KEGG pathways were significantly (padj < 0.05) enriched for predicted 3,109 target genes of up-regulated and 295 target genes of down-regulated miRNAs, respectively. oar-miR-432 was one of the most up-regulated miRNAs between Hu sheep and Tibetan sheep. And SIRT1 is one of the potential target genes of oar-miR-432. Furthermore, functional validation using the dual-luciferase reporter assay indicated that the up-regulated miRNA; oar-miR-432 potentially targeted sirtuin 1 (SIRT1) expression. Then, the oar-miR-432 mimic transfected into preadipocytes resulted in inhibited expression of SIRT1. This is the first time reported that the expression of SIRT1 gene was regulated by oar-miR-432 in fat metabolism of sheep liver. These results could provide a meaningful theoretical basis for studying the fat metabolism of sheep.

## 1 Introduction

MicroRNAs (miRNAs) are a kind of small RNA, whose length is about 22 nt (nucleotide). Previous studies revealed that miRNAs have distinctive biological characteristics in proliferation, differentiation, metabolism, and disease (Lin et al., 2020). In animals and plants, miRNAs are involved in the regulation of post-transcriptional gene expression. miRNAs usually bind to the 3'UTR region of mRNA to inhibit the post-transcriptional translation of target genes and enhance the degradation or repress the translation of mRNAs (Rouleau et al., 2017). In Chinese indigenous sheep, sheep can be divided into short/thin-tailed sheep, long/thin-tailed sheep, short/fat-tailed sheep, long/fat-tailed sheep, and fat-buttock sheep, because of the degree of fat deposition along the tail vertebra and the length of the tail vertebra (Lu et al., 2020). Hu sheep (short/fat-tailed sheep) and Tibetan sheep (short/thin-tailed sheep) are two Chinese indigenous sheep breeds with different tail types. Tail fat is the main energy source for sheep migration, drought, and food deprivation (Luo et al., 2021). However, studies mainly focus directly on tail fat to study fat metabolism, which is not the main organ of fat metabolism (Zhou et al., 2017; Li et al., 2020). The liver is a primary organ of fat metabolism, fat metabolization in the liver is equally important to its metabolism in fat tissue. Triglyceride is one of the lipids mostly formed in the liver, whose metabolism is mainly controlled through liver parenchyma cells. And the degree of fat deposition in fat tissue depends on the fat flow in the liver for fat synthesis. (Carotti et al., 2020). There are differences in the liver of sheep with different tail types that can reflect the underlying mechanism of sheep fat metabolism.

With the development of high-throughput sequencing technology, miRNA-Seq has been widely used in the omics analysis of humans (Zheng et al., 2016), mice (Peng et al., 2013), chickens (Sikorska et al., 2021) and cows (Zhang et al., 2019; Chen et al., 2020) species. And researchers showed that miRNA has an important function in fat metabolism (Deng et al., 2020). Many studies have explored the role of miRNA in liver fat metabolism disease models to clarify the process of disease occurrence. In a non-alcoholic fatty liver disease (NAFLD) mouse model, Lin et al. identified that miR-29a not only made body weight gain decrease, but also the subcutaneous, visceral, and intestinal fat accumulation and hepatocellular steatosis (Jeon and Carr., 2020). In the non-alcoholic steatohepatitis (NASH) mouse model, inhibiting the expression of miR-21 decreased liver injury, inflammation, and fibrosis (SOARES et al., 2016). In a high-fat-induced mouse model, miR-378 targeted *AMPK* to promote the occurrence of liver fibrosis and inflammation (Lin et al., 2019). Meanwhile, researchers have analyzed the expression patterns of miRNA in the liver of pigs (Li et al., 2021) and cows (Liang et al., 2017) across periods. These studies represented a foundation for further understanding the molecular regulatory mechanisms of liver tissue fat metabolism.

Because there is a genetic difference between Hu sheep (short/fat-tailed sheep) and Tibetan sheep (short/thin-tailed sheep) in tail type, comparing their livers’ miRNA features may find miRNAs affecting the fat metabolism of Hu sheep (short/fat-tailed sheep) and Tibetan sheep (short/thin-tailed sheep). Our results could provide a theoretical basis for further study of the fat metabolism between different sheep breeds.

## 2 Matericals and methods

### 2.1 Tissue collection and sequencing

All animal experiments were approved by the Science Research Department of the Institute of Animal Sciences, Chinese Academy of Agriculture Sciences (IAS-CAAS). Ethical approval complied with the Animal Ethics Committee of the IAS-CAAS (No. IAS 2019-49). Samples of liver tissues were collected from three Hu sheep (short/fat-tailed sheep, Yongdeng, Gansu, China) and three Tibetan sheep (short/thin-tailed sheep, Yushu, Qinghai, China). Samples from Hu sheep are named HG1, HG2, and HG3, respectively. Samples from Tibetan sheep are named ZG1, ZG2, and ZG3, respectively. All sheep were males and slaughtered at age 1.5. All samples were frozen in liquid nitrogen in 1.5 mL RNase-free freezing tubes and stored at −80°C for use. Trizol (Invitrogen, Carlsbad, CA, United States) was used to extract total RNA. A NanoDrop2000 spectrophotometer (Thermo Fisher Scientific, Wilmington, MA, United States) was used to quantify RNA purity at 260 and 280 nm. Six libraries were constructed with a commercial sequencing provider: BGI (Mortazavi et al., 2008; Wang et al., 2009). An Agilent 2,100 Bioanalyzer (Agilent Technologies, Palo Alto, CA, United States) was used to examine the integrity of the library. All FASTQ sequencing files have been stored in the Sequence Read Archive (accession numbers PRJNA785102).

### 2.2 Sequence analysis

The cleaning of the raw data was performed based on: 1) poor quality sequencing reads, 2) reads with 5′ adaptors and without 3’ adaptors; 3) reads without insert segments; and 5) reads containing poly A; and 6) reads longer than 18 nucleotides. To ensure that each small RNA had a unique label, according to the order of possible ribosomal RNA, small conditional RNA, small nucleolar RNA, small nuclear RNA (snRNA), and transfer RNA sequences to annotate (Balaskas et al., 2020). The sheep reference genome Oar_v3.1 (https://www.ebi.ac.uk/ena/browser/view/GCA_000298735.1, accessed on 20 February 2021) and miRbase21.0 (http://www.mirbase.org, accessed on 20 February 2021) was used to map clean reads with Bowtie2 (Langmead et al., 2009).

### 2.3 MiRNA identification and differential expression analysis

MiRDeep2 software was used to predict novel miRNAs (Kern et al., 2020). The expression of miRNA was calculated by absolute numbers counting of molecules using unique molecular identifiers (Pflug and Haeseler., 2018). Moreover, the lengths of small RNAs (sRNAs) and the proportion of miRNAs were calculated. The “oar-miR-" and “novel_mir” terms identify known miRNAs and novel miRNAs, respectively. Hu sheep is set as a control, DESeq2 software was used to perform the differential expression analysis, in which the statistical significance was set at a fold discover rate (FDR) adjusted *p*-value (padj ≤0.05) by Benja-mini-Hochberg and |Log2Foldchange| > 0.5.

### 2.4 Target gene prediction of miRNAs and gene function enrichment analysis

Miranda (John et al., 2004) and RNAhybrid (Lin et al., 2022) were used to find more accurate targets of differentially expressed miRNA (DE miRNA). g: Profiler was used for genes function enrichment analysis, in which the statistical significance was set at a fold discover rate (FDR) adjusted *p*-value (padj ≤0.05) by Benjamini–Hochberg (Raudvere et al., 2019). There are 3,109 target genes of upregulated and 295 target genes of downregulated DE miRNAs were annotated with Gene Ontology (GO) (http://www.geneontology.org/, accessed on 19 January 2022) and the Kyoto Encyclopedia of Genes and Genomes (KEGG) (http://www.genome.jp, accessed on 19 January 2022), respectively.

### 2.5 Quantitative real-Time PCR

Steam-loop real-time qPCR was used to validate miRNA sequencing data from seven randomly selected miRNAs (oar-miR-432, novel_mir70, novel_mir21, nov-el_mir64, novel_mir58, oar-miR-19b, and oar-miR-29b). The total RNA of each sample was reversed transcribed with a miRNA 1st Strand cDNA Synthesis Kit. RT-qPCR was performed on a LightCycler^®^ 480II qPCR system using miRNA universal SYBR qPCR Master Mix (Vazyme, Nanjing, China). U6 was used as the reference gene. To detect the expression of *SIRT1*, HiScript III 1st Strand cDNA Synthesis Kit (+gDNA wiper) and ChamQ universal SYBR qPCR Master Mix (Vazyme, Nanjing, China) were used. And *beta-actin* was used as the reference gene. The reverse transcription and PCR primer sequences are listed in Supplementary Table S1. The relative expression levels of miRNA and mRNA were calculated using 2^−ΔΔCT^ (Rao et al., 2013).

### 2.6 Dual -Luciferase reporter assay

To verify the target relationship of *SIRT1* and oar-miR-432, Xho I and NotI restriction enzyme cutting sites were amplified with the wild-type 3'UTR of the *SIRT1*. The primers are listed in Supplementary Table S1. The wild-type 3'UTR of the *SIRT1* was ligated to vectors and named psiCHECK2-*SIRT1*-3'UTR-WT.

Using a Site-Directed Mutagenesis Kit (Thermo Fisher Scientific, MA, United States), the mutant-type 3'UTR of *SIRT1* was obtained and named psiCHECK2-*SIRT1*-3'UTR-MT. PsiCHECK2-*SIRT1*-3'UTR-WT, psiCHECK2-*SIRT1*-3'UTR-MT, or pure vectors were co-transfected with oar-miR-432 mimics; pure vectors were co-transfected with negative control (NC) or oar-miR-432 mimics into 293T (Pan et al., 2018). After incubation for 6 h, the culture medium was changed. After 48 h of incubation, the relative luciferase activity in the cells was measured using a Dual-Luciferase Reporter Assay System (Promega, Promega, WI, United States). Each treatment was performed 4 times for each group. All plasmid, oar-miR-432 mimics, and negative control were synthesized by GenePharma (Shanghai, China).

### 2.7 Sheep preadipocytes culture and transfection

Sheep preadipocytes were isolated from the tail fat of a 70-day-old Hu sheep fetus by collagenase digestion. Preadipocyte transfection and culture were according to our previous method (Jin et al., 2022). When the cell showed contact inhibition, we collected cells and extracted protein.

### 2.8 Western blot

Proteins from cell were extracted with RIPA buffer and separated on SDS-PAGE gel including 4% concentrated glue and 12% separation gel. After transfer, the PVDF blot membranes were blocked and then probed with rabbit polyclonal antibody against SIRT1 (1: 1,000, Proteintech, Chicago, IL, United States) at 4°C overnight. Alpha-tubulin poly-clonal antibody (1:3,000, Abclonal, Beijing, China) was used as an internal reference. These blots were further conjugated with a goat anti-rabbit IgG secondary antibody (1:1,000, Proteintech, Chicago, IL, United States) labeled with HRP *via* incubation and revealed with an ECL kit (Engreen, Beijing, China), and exposed to X-ray films. Blot intensity quantification was performed using ImageJ software (1.51j8) (Rha and Gyeol Yoo, 2015).

### 2.9 Statistical analysis

The data were processed by SPSS 20.0 two-tailed Student’s t-test (Singh et al., 2019). All the results are presented as means ± standard deviation. Furthermore, * indicates statistically significant (*p* < 0.05). ** indicates statistically significant (*p* < 0.01).

## 3 Result

### 3.1 Quality control

The results of the miRNA-Seq data after quality control are displayed in Table 1. The clean tag count of each sample ranged from 27 to 28 million, and the Q20 of clean tags ranged from 98.20% to 98.50%. About 88.63%–92.75% of the clean reads were mapped to the sheep reference genome.

**TABLE 1:** Summary of sequencing data for each library.

Sample name	Sequence type	Raw tag count	Clean tag count	Percentage of clean tag (%)	Q20^*^ of clean tag (%)	Percentage of mapped tag (%)
HG1 (short/fat-tailed sheep)	SE50	28,376,193	27,508,714	96.94	98.50	92.75
HG2 (short/fat-tailed sheep)	SE50	28,289,347	27,054,271	95.63	98.40	91.58
HG3 (short/fat-tailed sheep)	SE50	29,793,809	28,483305	95.60	98.40	90.48
ZG1 (short/thin-tailed sheep)	SE50	30,184,839	28,487,066	94.35	98.30	88.63
ZG2 (short/thin-tailed sheep)	SE50	28,886,721	27,154,416	94.70	98.20	89.46
ZG3 (short/thin-tailed sheep)	SE50	29,008,123	27,666,601	95.38	98.50	89.77

### 3.2 Identification of miRNAs

In this study, 134 known miRNAs and 275 novel miRNAs were identified from HG1; 132 known miRNAs and 291 novel miRNAs were identified from HG2; 137 known miRNAs and 298 novel miRNAs were identified from HG3; 132 known miRNAs and 295 novel miRNAs were identified from ZG1; 133 known miRNAs and 198 novel miRNAs were identified from ZG2; and 129 known miRNAs and 273 novel miRNAs were identified from ZG3 (Supplementary Table S2).

### 3.3 Analysis of differentially expressed miRNAs

**FIGURE 1:**
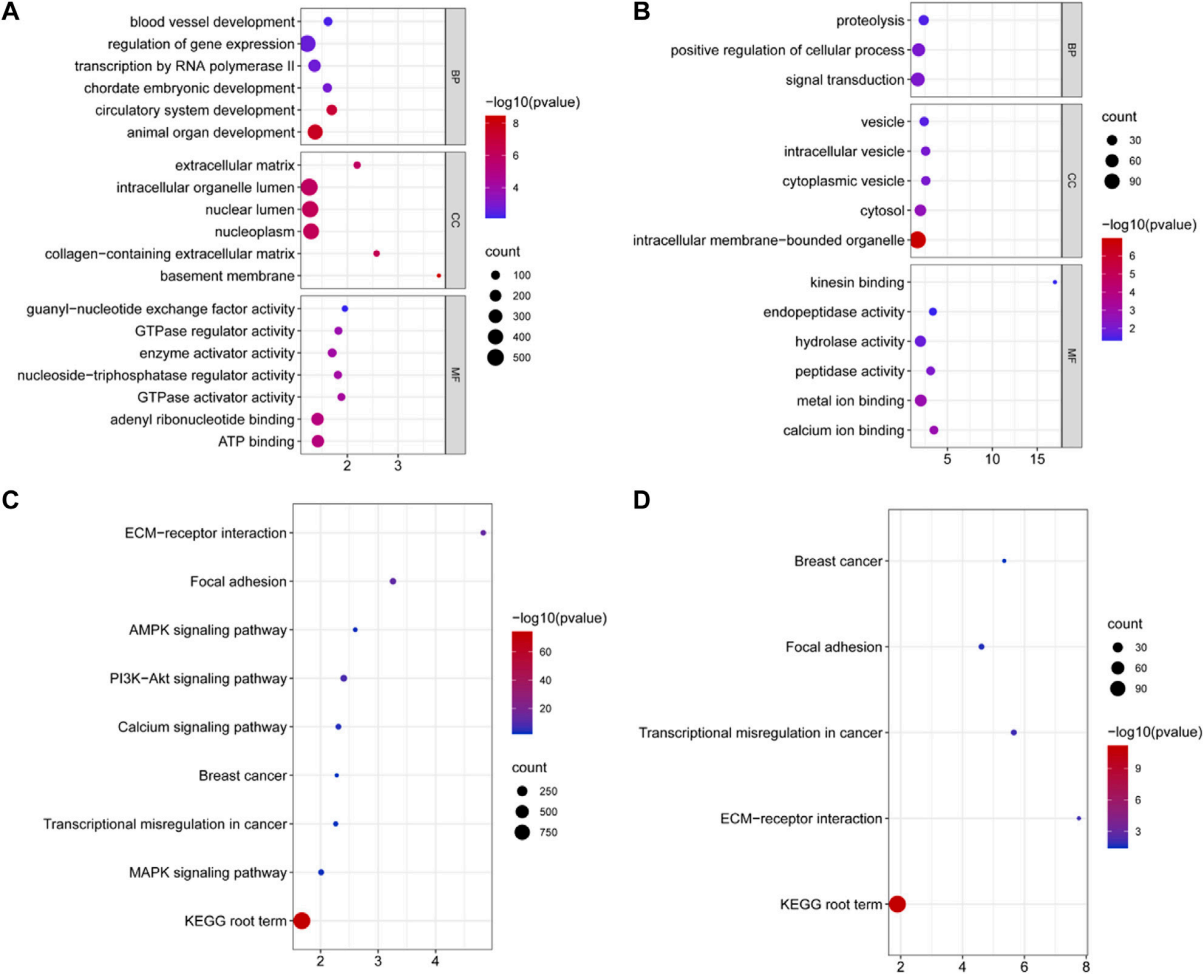
The volcano plots of all expressed miRNAs in the livers of Hu sheep (short/fat-tailed sheep) and Tibetan sheep (short/thin-tailed sheep). The *x*-axis denotes the values of log2 (fold-change), whereas the *y*-axis denotes the −log10 (padj). The colored dots represent the expressed miRNAs, with blue indicating downregulated miRNAs and red indicating upregulated miRNAs (padj ≤0.05). The black dots indicate that the miRNAs are not statistically significant (padj >0.05).

We found 379 novel miRNAs and 139 known miRNAs. Hu sheep is set as a control, based on the padj ≤0.05, we detected 11 DE miRNAs in ZG compared with HG (Figure 1 and Supplementary Table S3). There are six upregulated miRNAs, including novel_mir471, oar-miR-432, novel_mir21, novel_mir59, novel_mir394 and, novel_mir70. There are five downregulated miRNAs, including oar-miR-29b, novel_mir58, novel_mir54, oar-miR-19b, and novel_mir64. Three miRNAs were reported that were associated with fat metabolism.

### 3.4 DE miRNAs target prediction and functional analysis

**FIGURE 2:**
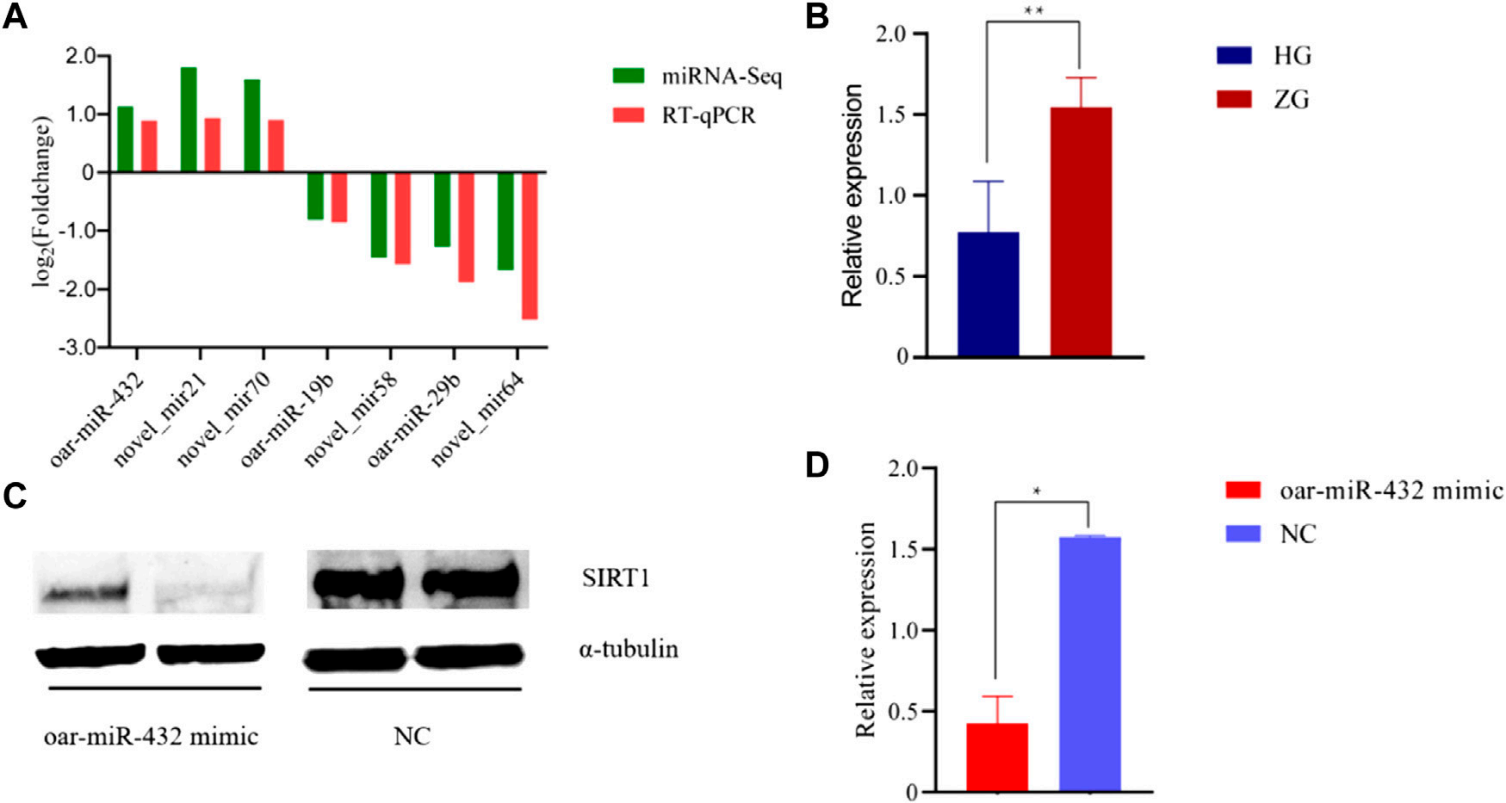
Significantly enriched Gene Ontology and KEGG for the target genes of DE miRNAs. **(A)** Some GO terms of target genes of upregulated DE miRNAs for BP, CC, and MF in two groups. **(B)** GO terms of target genes of downregulated DE miRNAs for BP, CC, and MF in two groups. The *x*-axis displays enrichment, and the *y*-axis rep-resents the GO terms. The filled colored circles display each statistically significant GO term. The size of the circles represents the gene number. **(C)** Signal pathway of the target genes of upregulated DE miRNAs in two groups. **(D)** Some signal pathways of the target genes of upregulated DE miRNAs in two groups. The *x*-axis displays the enrich-ment factor of the target genes, and the *y*-axis represents the KEGG pathway. The filled colored circles represent each statistically significant KEGG pathway. The size of the circles represents the number of genes.

Miranda and RNAhybrid software were used to predict the target genes of DE miRNAs, resulting in 3,404 predicted target genes (Supplementary Table S4). GO annotation enrichment was used to describe the functions of the target genes of upregulated and downregulated DE miRNAs. These were involved in cellular components (CCs), molecular function (MF), and biological processes (BP), including animal organ development, intracellular organelle lumen, ATP binding, intracellular vesicles, and kinesin and calcium ion binding (Figures 2A,B and Supplementary Table S5). A total of 115 GO terms were significantly enriched by target genes of the upregulated DE miRNAs, and 54 terms were significantly enriched by target genes of the downregulated DE miRNAs. DE miRNAs were used in a KEGG pathway enrichment analysis. Based on all the target genes of upregulated and downregulated miRNAs, 67 and 5 KEGG pathways were significantly enriched, respectively (Supplementary Table S6). As shown in Figures 2C,D, the ECM–receptor interaction signaling pathway, KEGG root term signaling pathway, transcriptional regulation in the cancer signaling pathway, the focal adhesion signaling pathway, and the breast cancer signaling pathway were simultaneously enriched. Other signaling pathways related to fat metabolism were enriched, including the PI3K-Akt signaling pathway, calcium signaling pathway, AMPK signaling pathway, and MAPK signaling pathway, which are related to fat metabolism.

### 3.5 Verified the DE miRNA and the expression of miRNA by RT-qPCR

**FIGURE 3:**
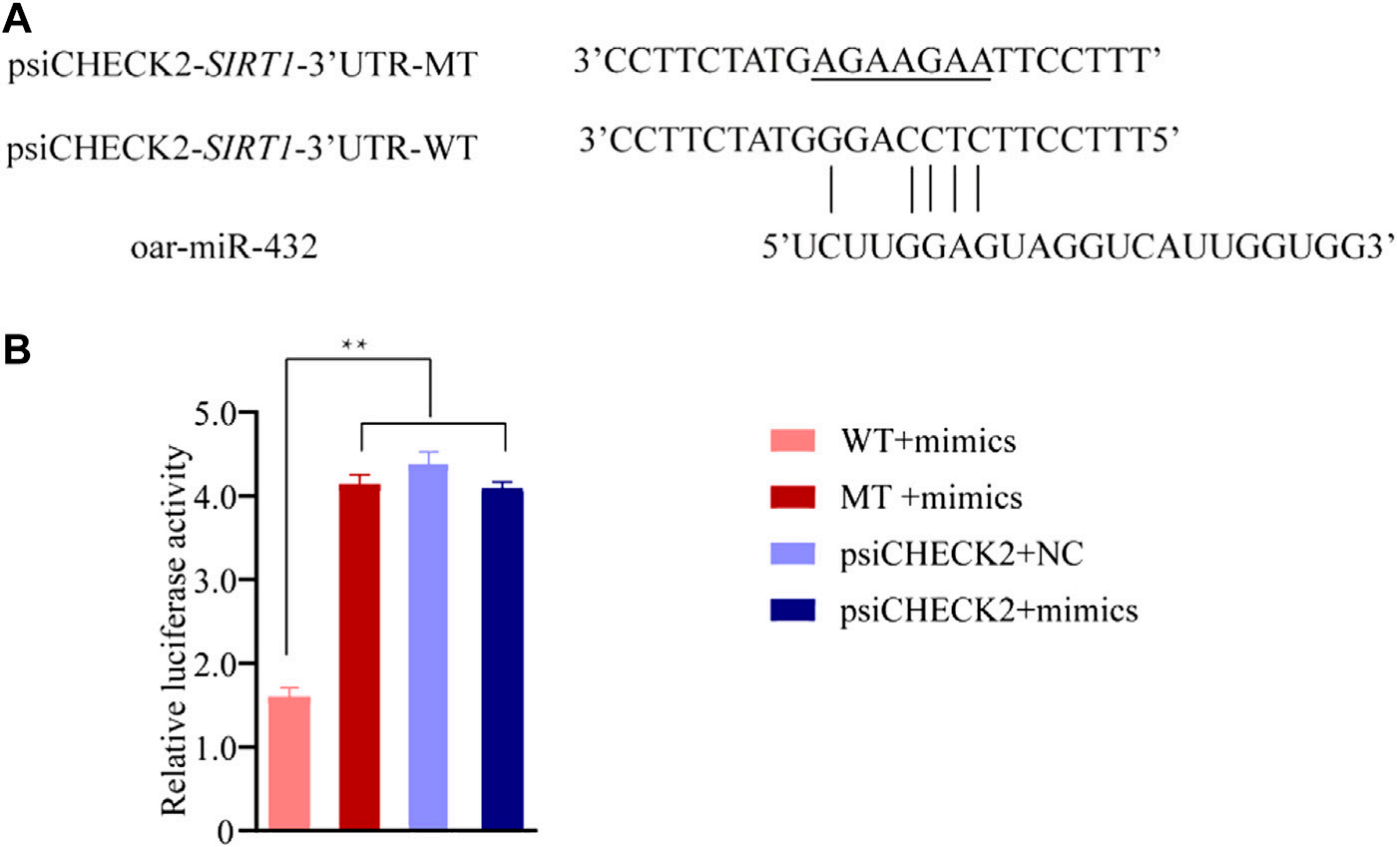
The results of RT-qPCR and Western blot. **(A)** RNA-Seq and RT-qPCR results of seven differentially expressed miRNAs in ZG compared with HG. **(B)** RT-qPCR results of SIRT1 in HG and ZG. **(C) (D)** Western blot results of SIRT1 in preadipocytes. NC exhibits negative control.

The RT-qPCR technique was used to validate the sequencing results. Seven miRNAs were randomly selected for RT-qPCR verification. The validation results are displayed in Figure 3A and Supplementary Table S7.

### 3.6 Plasmid identification

Eight randomly selected monoclonals and vector universal primers were used to identify the wild-type psiCHECK2 plasmid by polymerase chain reaction (PCR) (Supplementary Figure S1) and sequencing. The sequencing primers are shown in Supplementary Table S1. Site-directed mutation was used to obtain the mutant-type psiCHECK2 plasmid. The sequencing results of wild-type psiCHECK2 plasmid and mutant-type psiCHECK2 are in Supplementary Table S8 and Supplementary Table S9. Eventually, the plasmids were constructed successfully.

### 3.7 Validation of the target relationship between oar-miR-432 and *SIRT1*

**FIGURE 4:**
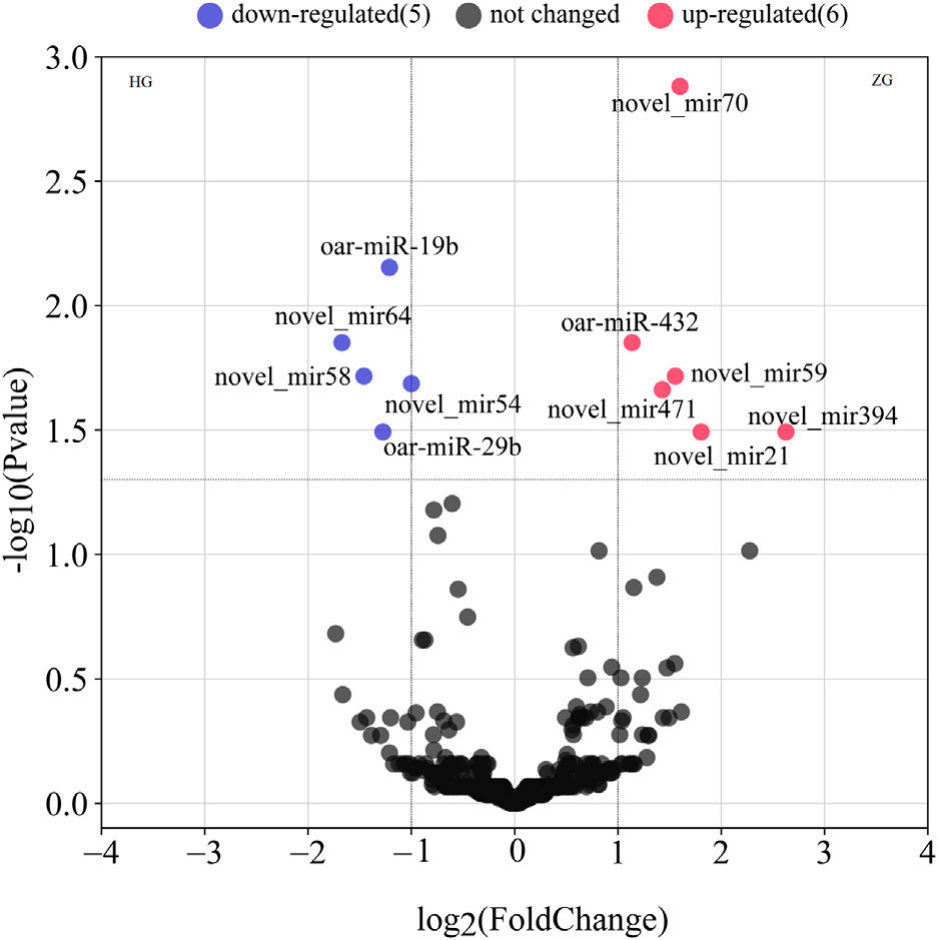
Result of the luciferase reporter assay. **(A)** Potential binding site between oar-miR-432 and SIRT13'UTR. The underlined sequences represent the mutant sites. **(B)** WT exhibits the psiCHECK2-SIRT1-3'UTR-WT. MT exhibits psiCHECK2-SIRT1-3'UTR-MT. psiCHECK2 exhibits psiCHECK2 pure vectors. Mimics exhibits oar-miR-432 mimics. NC exhibits negative control. **: indicates statistically significant (*p* < 0.01).

A dual-luciferase reporter assay indicated that oar-miR-432 significantly suppressed the luciferase activities for co-transfection with *SIRT1* 3'UTR wild-types, although did not affect the mutant types of SIRT1 3'UTR or blank vectors (Figure 4B and Supplementary Table S10). These results initially confirmed the direct interactions between oar-miR-432 and *SIRT1*.

### 3.8 Expression of *SIRT1* in Liver tissue

The RT-qPCR results showed that the expression trends in oar-miR-432 and *SIRT1* were contrasting. oar-miR-432 was highly expressed in the liver tissue of Hu sheep, while the *SIRT1* was highly expressed in the liver tissue of Tibetan sheep (Figure 3B, Supplementary Table S7).

### 3.9 Expression of *SIRT1* in preadipocytes

Oar-miR-432 mimics and negative control were transfected into preadipocytes. Then we detected the expression of oar-miR-432 and SIRT1*.* The expression of oar-miR-432 was increased by oar-miR-432 mimics (Jin et al., 2022). The result of the Western blot showed the expression of SIRT1 was inhibited by oar-miR-432 mimics (Figures 3C,D, Supplementary Table S11, Supplementary Figure S2, Supplementary Figure S3).

## 4 Discussion

Thus far, miRNA expression has been studied in the liver tissues of buffalos (Rha and Gyeol Yoo, 2015), dairy cows (Bu et al., 2017), mice (Seclaman et al., 2019), rats (Wang et al., 2017), pigeons (Wang et al., 2020), pigs (Kai et al., 2019), chickens (Xu et al., 2019), and geese (Zheng et al., 2015). RNA-Seq was used to construct 41 pairs of ceRNA networks on liver tissue from three Holstein cows, which provide new insight into resolving bovine lipid metabolism (Liang et al., 2017). In bovine hepatocytes, miR-27a-5p inhibited calcium sensing receptor (*CASR*) expression, triacylglycerol (*TAG*) accumulation was significantly suppressed, and low very density lipoprotein (*VLDL*) secretion was reduced (Yang et al., 2018). established miRNA-mRNA regulatory networks related to lipid deposition and metabolism in the livers of Landrace pigs with the extreme backfat thickness (Kai et al., 2019). RNA-Seq was used to construct miRNA-mRNA networks between Jinhua and Landrace pigs (Huang et al., 2019). These studies provided new insights into the molecular mechanisms to explore fat metabolism in pigs. Also, the study found there was a lncRNA-FNIP2/miR-24-3p/FNIP2 axis, which can regulate lipid metabolism in Sanghuang chicken liver (Guo et al., 2021).

In this study, we used high-throughput sequencing to identify the expression of miRNA in the livers of Hu sheep and Tibetan sheep. This study complements the current understanding of miRNA expression patterns in sheep livers and will help future research on the specific role of miRNA in regulating fat metabolism. In our study, we identified 11 differential miRNAs. miR-432, miR-19b, and miR-29b are associated with fat metabolism, and a previous study showed that miR-432 inhibits milk fat synthesis by targeting stearoyl CoA desaturase (*SCD*) and *LPL* in ovine mammary epithelial cells. Additionally, miR-432 inhibits the proliferation of ovine mammary epithelial cells (Hao et al., 2021). Transcriptome analysis revealed that miR-432 was differentially expressed in the backfat of cattle; the protein kinase AMP-activated catalytic subunit alpha 1/2 (*PRKAA1/2*) and peroxisome proliferator-activated receptor alpha (*PPARA*) were regulation targets to modulate lipid and fatty acid metabolism (Sun et al., 2014). Interestingly, miR-432 was differentially expressed in tail fat between Hu sheep and Tibetan sheep, which could have an important function in sheep fat metabolism (Fei et al., 2022). In mice SVF cells, miR-19b had an inhibitory effect on the browning process of adipose tissue (Lv et al., 2018). Researchers found that miR-29b can regulate blood sugar in adult mice, representing a target for treating metabolism disease (Hung et al., 2019). Additionally, miR-29b inhibits the differentiation of pig muscle and subcutaneous preadipocytes through targeted regulation complement component 1 (*C1q*) and TNF-related protein 6 (*CTRP6*) (Wu et al., 2021). Ma et al. found that lncRNAs, including TCONS_00372,767 and TCONS_00171,926, were related to fat metabolism among Lanzhou fat-tailed sheep, small-tailed Han sheep, and Tibetan sheep, and constructed two co-expression networks of differentially expressed mRNA and lncRNA (Ma et al., 2018). The research conducted by Cheng et al. showed that there were differences in the livers of Mongolian and Lanzhou fat-tailed sheep through RNA-Seq, which provided a reference for researching the sheep genome (Cheng et a., 2016).

Hu sheep set as a control to identify DE miRNAs. The extracellular matrix (ECM)–receptor interaction signaling pathway was significantly enriched by the target genes of upregulated DE miRNAs and downregulated DE miRNAs. The main constituents of the ECM–receptor interaction signaling pathway in adipose tissue include collagen (type I, IV, and VI), fibronectin (FN), laminin (LN1,8), hyaluronan, and proteoglycan (Lee et al., 2013). The functional analysis showed differently expressed genes in the subcutaneous and intramuscular fat of cattle were enriched in ECM–receptor interaction signaling pathway. In the study of San et al., some genes which affected intramuscular fat (IMF) deposition was significantly enriched in the ECM–receptor interaction signaling pathway (San et al., 2021). In our study, the target genes of upregulated DE miRNAs were enriched in the PI3K-Akt signaling pathway, calcium signaling pathway, the AMPK signaling pathway, and MAPK signaling pathway, which are associated with fat metabolism (Fu et al., 2022). In our study, forkhead boxO3 (*FoxO3*) was enriched in the PI3K/AKT signaling pathway and AMPK signal pathway. In mice fed high-glucose and high-sucrose diets, FoxO3 promoted hepatic triglyceride synthesis and hepatic triglyceride accumulation in the liver by positively regulating the sterol regulatory element binding transcription factor 1 (*SREBP1c*) (Wang et al., 2019). Additionally, *SIRT1* was enriched in the AMPK signal pathway. SIRT1 plays an important biological role in regulating liver lipid metabolism, oxidative stress, and inflammation, and can be used as a therapeutic target for the treatment of alcoholic and non-alcoholic fatty liver diseases (Ding et al., 2017). It has been shown that vitamin D can activate the AMPK/SIRT1 pathway to inhibit the accumulation of fat in C2C12 skeletal muscle cells (Chang and Kim., 2019). miR-29 can regulate SIRT1 to inhibit fat deposits in mouse livers (Kurtz et al., 2015). Additionally, Liang et al. that dietary cholesterol can promote the occurrence of steatohepatitis through the calcium signaling pathway (Liang et al., 2018). In a diabetic mouse model, the ginsenoside metabolite compound K inhibits the activation of the NLR family pyrin domain containing 3 (*NLRP3*) through the NF-κB/p38 signaling pathway (Song et al., 2018). Previous studies have shown that in human liver fat cells, transforming growth factor-beta 1 (*TGF-β1*) regulates the platelet-derived growth factor receptor beta (*PDGFD-β*) subunit to maintain the activation and proliferation of fat cells (Pinzani et al., 1995). In our previous study, these pathways were enriched significantly, including ECM–receptor interaction signaling pathway, PI3K-Akt signaling pathway, calcium signaling pathway, AMPK signaling pathway, and MAPK signaling pathway (Fei et al., 2022). All of the results showed that these pathways could have a vital function in sheep fat metabolism.

In this research, our goal was to preliminarily determine how oar-miR-432 and *SIRT1* regulate fat metabolism. In our current study, we use dual-luciferase reporter assays to verify the binding relationship between miR-432 and the target gene *SIRT1*. The expression of *SIRT1* was detected in the liver tissues of Hu sheep and Tibetan sheep. RT-qPCR results showed that the expression of SIRT1 in Tibetan sheep was significantly higher than that in Hu sheep. We transfected oar-miR-432 in preadipocytes, and we found oar-miR-432 can inhibit the expression of SIRT1 at the protein level. This is the first time reported that the expression of *SIRT1* gene was regulated by oar-miR-432 in fat metabolism of sheep liver. The regulation of the process leading from mRNA to protein is generally very complex. Studies have shown that gene repression could be changed due to the post-transcriptional regulation of miRNA (Pasquier and Gardès., 2016). Our study showed that oar-miR-432 downregulated the expression of *SIRT1* at the transcriptional level in sheep liver tissue. Meanwhile, the result of Western blot showed that oar-miR-432 can downregulated the expression of SIRT1 protein in preadipocytes. Our study indicated that p53 is independent of the oar-miR-432 *SIRT1* gene regulation.

## 5 Conclusion

In summary, our results provide a comprehensive expression profile of miRNA in the livers between two different sheep breeds. The DE miRNAs reported in this article may play an important role in sheep fat metabolism. We have verified that oar-miR-432 can target the regulation gene *SIRT1* in sheep. This study provides a reference for further research addressing the modulation of fat metabolism in different sheep breeds.

## Data Availability

The datasets presented in this study can be found in online repositories. The sequencing files have been stored in the Sequence Read Archive (accession numbers PRJNA785102).
