# Supplementary material for: MiRNA-Seq reveals key MicroRNAs involved in fat metabolism of sheep liver
Source: Front Genet. 2023 Mar 9;14:985764. doi: 10.3389/fgene.2023.985764 (PMC10035661; doi:10.3389/fgene.2023.985764)
Supplement: Supplementary file 4 [file Table12.DOCX]

Supplementary Material

# Supplementary Figures


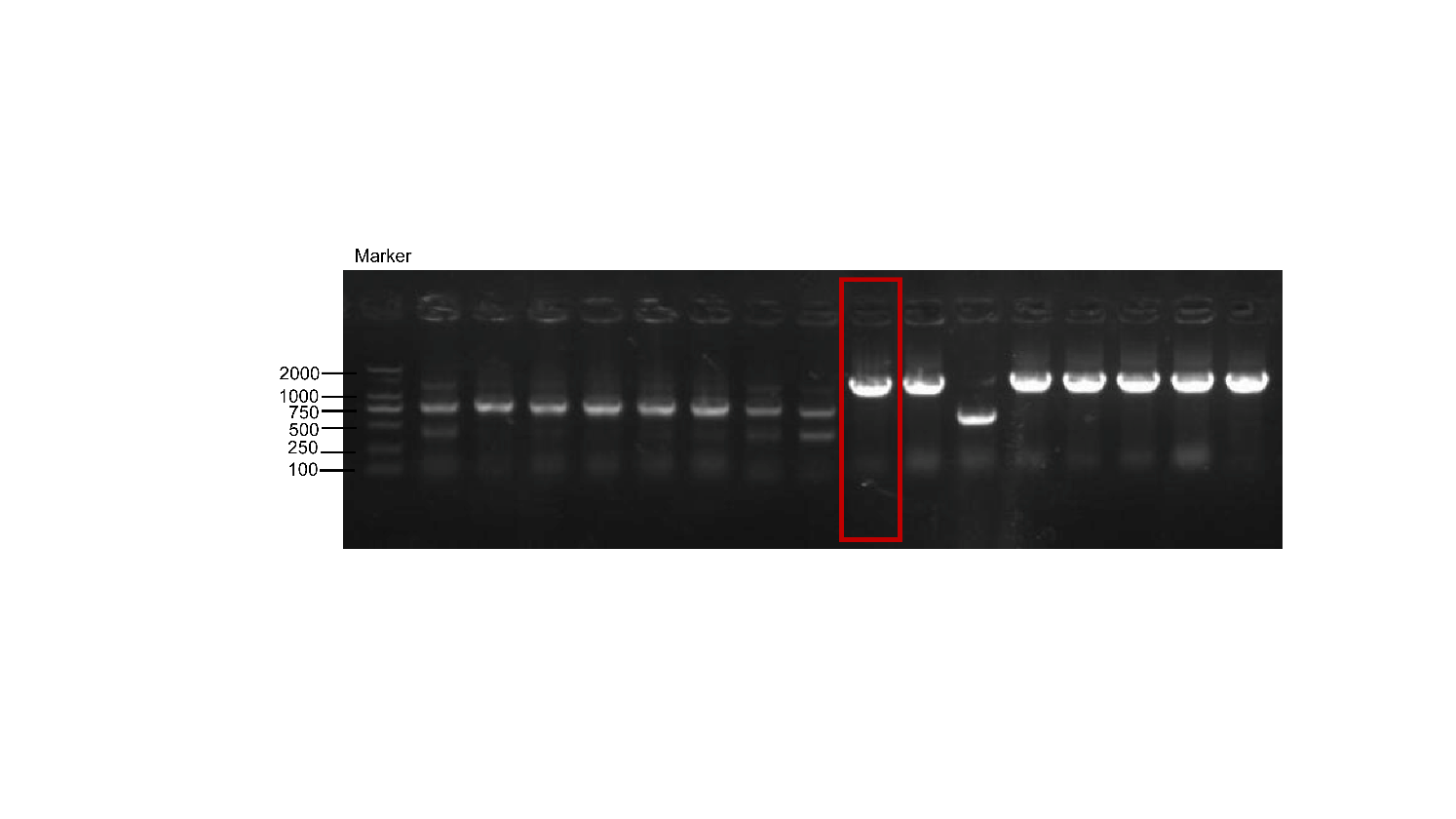


**Supplementary Figure 1.** The positive clones identified by PCR are marked with a red frame.


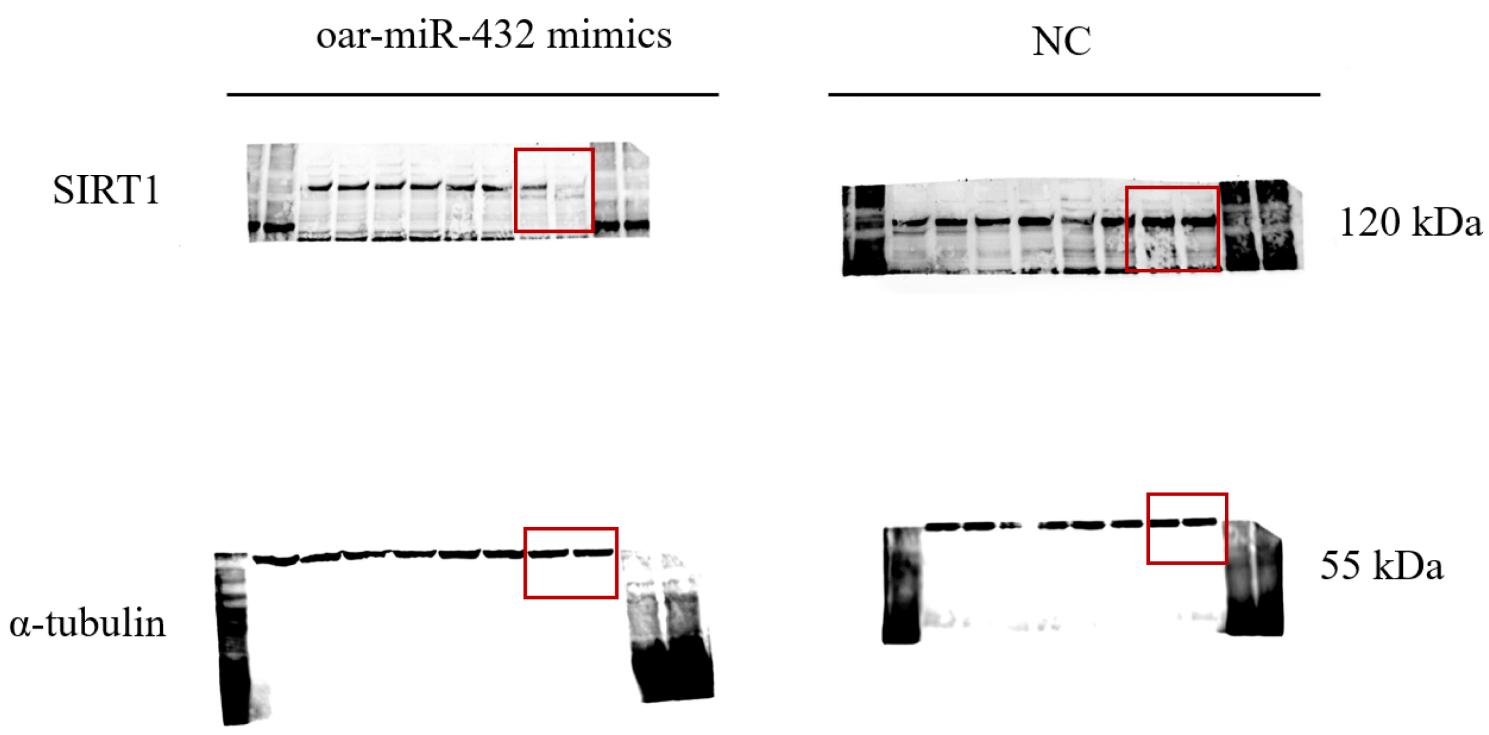


**Supplementary Figure 2.** The original protein result of SIRT1 in sheep preadipocytes. When cell showed contact inhibition, the expression of SIRT1 in sheep preadipocytes are marked with a red frame.


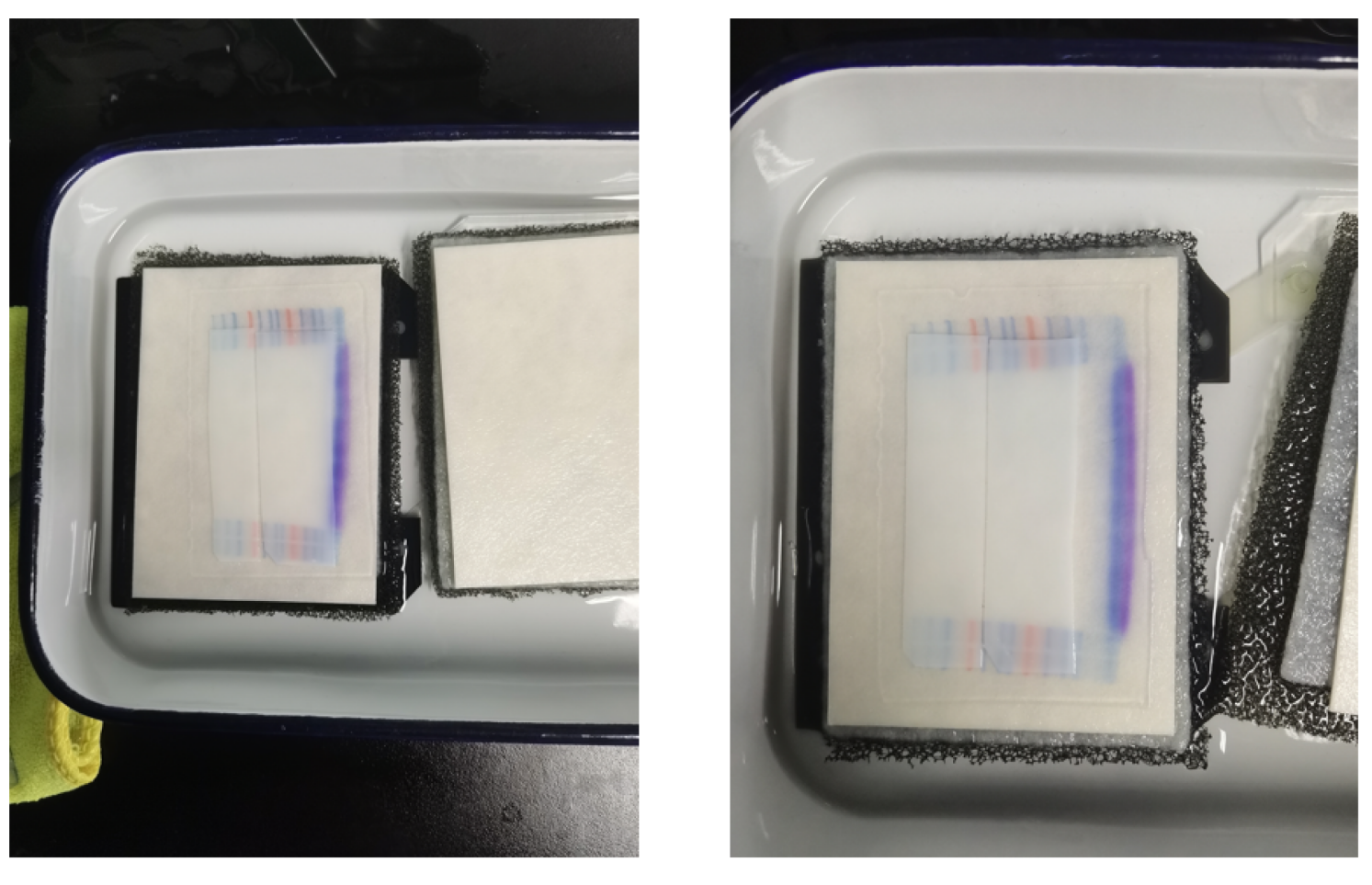


**Supplementary Figure 3.** Process diagram of SIRT1 protein expression in sheep preadipocytes.
