# Supplementary material for: MiRNA-Seq reveals key MicroRNAs involved in fat metabolism of sheep liver
Source: Front Genet. 2023 Mar 9;14:985764. doi: 10.3389/fgene.2023.985764 (PMC10035661; doi:10.3389/fgene.2023.985764)
Supplement: Supplementary file 5 [file Table9.DOCX]

NNNNNNAGNATGANGTAGTACATGCAAGAGCTTCGTGGAGCGCGTGCTGAAGAACGAGCAGTAATTCTAGGCGATCGCTCGAGATCCAGCTAGGACCATTACTGCCAGAGAAAAAGATTTTATTGAATGGCCATTTCCCTACCTAAAAGATGTTTCAATCTGAATTTGACTACACTAAAGAATGCAGTATATTTAGTTTTCCATTTGCATGATCTGTGTGTGTGCTATAGATGATATTTTAAATTGAAAAATTTGTTTTAAATTATTTTTACAGTGAAGACTGTTTCCAGCTCTTTTTATATTGTACATAGTCTTTTATGTAATCTACTGGCATATGTTTTGTAGACTGTTTAATGACTGGATATCTTCCTCAATCTTTTGAAATACAGAACCAGTGTTTTATACTTGTACACTGTTTTAAGTCTATTAAAAGTGTCATTTGACTTTTTTTCTGTTAGCTTACACTGTTTAAGGTAAAAACTTTAAAAATTGTGCAGCTTTTGTACAGCTTTGAGGGCTATTAGATGCTACATTTTTTTTTTCAGTTTATATACAAGTTAGTACTAAAATAGTACTAAAAGTGCTGGTTGTAGTAACTGGTGAAAGTGAAGTCAGTCGTGTCCAACTGTTTGCGACCCCATGGACTGTAGCCTATCAGGCTCCTCCGTCCATGGGATTTTCTAGGCAAGAGTGCTGGAGTGGATTGCCATTTCCTTAGAAGAAGTATCTTCCTGACCCAGGAATCAAACCTGGGTCTCCTGCATTGCAGGCAGACGCTTTACCATCTGAGCCACCAGGGAAGCTCCTGTACTAACTGTACATCACCAAAATGTCCTGTTCTAGACCAGGAGCCTTCCCCCCTCTCACTAACCCTGCCTAAGAATAACTGGGAAGGTTGAAGCAAGAGGACGAGGAGGCAGCAAGGACGAGATCTGATCAGCAGCAGACCTGACAGGTCCGGAGATACTGAGGACAGAAGGACCTGCATGCTGAGTCCATGGGTGCAGAGTTGNTATGACATGACCTGATGAACTTCGGAGTTGNGATGGACAGGAGCCTGCATG
